# Path Planning and Motion Control of Indoor Mobile Robot under Exploration-Based SLAM (e-SLAM)

**DOI:** 10.3390/s23073606

**Published:** 2023-03-30

**Authors:** Rohit Roy, You-Peng Tu, Long-Jye Sheu, Wei-Hua Chieng, Li-Chuan Tang, Hasan Ismail

**Affiliations:** 1 Department of Mechanical Engineering, National Yang Ming Chiao Tung University, Hsinchu 30010, Taiwan; rohitroy41.en08@nycu.edu.tw (R.R.); you.peng.tu.0302@gmail.com (Y.-P.T.); newton4538.eo85g@nctu.edu.tw (L.-C.T.); 2 Department of Mechanical Engineering, Chung Hua University, Hsinchu 30012, Taiwan; ljsheu@chu.edu.tw; 3 Jurusen Teknik Mesin, Universitas Negeri Malang, Malang 55165, Indonesia; hasan.ismail.ft@um.ac.id

**Keywords:** motion planning, vehicle control, mobile robot, LiDAR localization

## Abstract

Indoor mobile robot (IMR) motion control for e-SLAM techniques with limited sensors, i.e., only LiDAR, is proposed in this research. The path was initially generated from simple floor plans constructed by the IMR exploration. The path planning starts from the vertices which can be traveled through, proceeds to the velocity planning on both cornering and linear motion, and reaches the interpolated discrete points joining the vertices. The IMR recognizes its location and environment gradually from the LiDAR data. The study imposes the upper rings of the LiDAR image to perform localization while the lower rings are for obstacle detection. The IMR must travel through a series of featured vertices and perform the path planning further generating an integrated LiDAR image. A considerable challenge is that the LiDAR data are the only source to be compared with the path planned according to the floor map. Certain changes still need to be adapted into, for example, the distance precision with relevance to the floor map and the IMR deviation in order to avoid obstacles on the path. The LiDAR setting and IMR speed regulation account for a critical issue. The study contributed to integrating a step-by-step procedure of implementing path planning and motion control using solely the LiDAR data along with the integration of various pieces of software. The control strategy is thus improved while experimenting with various proportional control gains for position, orientation, and velocity of the LiDAR in the IMR.

## 1. Introduction

Autonomous vehicle technology has been emerging for more than the last two decades. This emerging technology has been enabled by major theoretical progress in the computational aspects of mobile robot motion planning and feedback control theory implemented in vehicles. Research efforts have undoubtedly been spurred by the expectation that autonomous vehicles will become a reality on our roads in the near future. Many companies aim to deliver systems for autonomous driving achieving SAE Level 5. Thus, it runs much more complex software than typical premium cars of today, which requires recorded data supported by onboard and surrounding sensors [1].

Moreover, the navigation problem is the main issue of autonomous vehicles that the researchers focus on. The autonomous vehicles and robot navigation problem is divided into localization problems and path planning and control problems. A global positioning system (GPS) is applied to the outdoor environment to achieve vehicle localization and navigation. However, the indoor environment requires a completely different methodology for vehicle localization and navigation because of the absence of satellite positioning signals [2].

Simultaneous localization and mapping (SLAM) is the essential part that allows autonomous or driverless vehicles to build a map and localize themselves on that map simultaneously. SLAM algorithms allow the vehicle to map out unknown environments and subsequently use the map information to carry out tasks such as path planning and obstacle avoidance. SLAM has been widely researched, and many different variations have been proposed. It can be classified into filter-based and optimization-based approaches [2,3]. It maintains the information about the environment and the states of the robot as a probability density function. On the other hand, the global optimization approach, which is based on saving some keyframes in the environment and uses bundle adjustment to estimate the motion [4], is currently a popular approach for vision-based SLAM such as ORB-SLAM [5,6,7] and also Google’s cartographer [8,9]. In addition to solving SLAM problems, the convolutional neural network SLAM which is currently available as RatSLAM is proven to have a superior performance in some situations [10,11].

Subsequently, the next problem in autonomous technology is how the robots and vehicles use the rich environment information from that perceptual equipment to find a proper path to the target position, follow the planned path, and avoid obstacles simultaneously. Accordingly, Muraleedharan et al. [12] studied the behavior control of an autonomous vehicle which is distinctly divided into two tasks: path planning and path following, which are alternatively executed. Planning has to be repeated since the planner must consider safety constraints such as collision avoidance and speed limitation. Moreover, path following is in addition to the basic target of path planning for keeping a lane, overtaking, etc.

Generally, computational complexity, local optimum, and adaptability are the main difficulties for robot path planning problems [13]. Thus, a typical hierarchy of the decision-making problems in current autonomous driving systems is structured into route planning, behavioral decision-making, local motion planning, and feedback control, where the solution of one problem is the input to the next [14]. Since SLAM is often combined with exploration, in some active SLAM methods, the robot movements switch between exploration and SLAM tasks in a discrete way depending on the uncertainty criterion value [15].

Many path planning methods have been explored in different approaches to obtain autonomous ability [16,17,18,19,20,21,22,23,24]. Some approaches in the literature [25,26,27,28] are developed under a robot operating system (ROS), offering a core set of software for operating robots that can be extended according to the prebuilt libraries and packages. In particular, LiDAR and odometer information collection are combined to solve the indoor robot SLAM and path planning technology problems by Wei et al. [27]. In order to improve the robot’s capability of path planning in a dynamic environment, Cheng, Li, and Liu [29] adopted the dynamic window approach (DWA) platform for the local obstacle avoidance method. The algorithm samples several sets of velocities in the velocity space composed of linear and angular velocities and simulates the robot’s trajectory at these velocities over time. After obtaining multiple sets of trajectories, they are scored, and the best one is selected as the actual motion of the robot. A similar approach [30] is introduced in which the path re-planning algorithm based on a topology map not only solves the failure of local path planning due to the limited range of the local map, but also simplifies the process and reduces resource consumption by SLAM. Zhang et al. performed a path planning method combining the A* algorithm for global paths and the DWA algorithm for local paths using mobile robots for indoor rescue [31]. A sensor fusion method has been studied using stereo vision and LiDAR by Moghdam, Sardha, and Feng [32]. They concluded that both laser and vision data are of utmost necessity for indoor path planning. A recent study from Zhao and Hwang using 2D LiDAR SLAM has demonstrated a small simulation using the main nodes of ROS for path planning [33]. However, no accounts of obstacle detection are mentioned in their research. All of the references use LiDAR, inertial measurement units (IMUs), and video cameras, which amount to more than one sensor, as their source for localization while motion planning.

In our previous study, described in [2], an exploration-based SLAM (e-SLAM) method is introduced for indoor mapping using only LiDAR. It meshes out vertical walls and trims the static furniture and the moving object from the same space of the IMR for the LiDAR mesh formation. LiDAR is used as the only sensor for active localization with LQE estimation for translational and rotational assistance. In this research, we extend our work using only LiDAR sensor data for path planning to find an optimized path to the target position and motion planning for the IMR to follow the planned path and avoid obstacles simultaneously. The novelty of this research adheres to the fact that LiDAR is the only sensor in the vehicle that is responsible for its active localization during exploration. The integrated image of the floor plan is then used to partition the plane into Voronoi segments to acquire an optimized path while also keeping in mind obstacle detection. The most minimal number of poses then is automatically selected on the Voronoi edges to compute and design a complete path for the vehicle. Exploration and minimum number of poses are given importance for path planning; hence, a generalized Voronoi algorithm is used in this research. However, the path planning algorithm is independent of the control strategy, and hence, it adheres to any planning algorithm keeping in mind the dimension of the IMR and obstacles having a height below 160 cm.

In this research, we present the potential of an automatic inter-office delivery robot using only LiDAR. With varied functionalities, given an initial floor plan, the Voronoi edges are first computed using only LiDAR cloud points, and then the path planning is performed. Special design methods in this manuscript are explained for its navigation. A better estimation method is constructed for the IMR control strategy. The current pose detected by the LiDAR itself is further used to plan the next path. In this research, we provide a step-by-step implementation integrating four sets of software, namely the PLC-based controller platform WINPC32, the EtherCAT motor driver, the path planning software Sidomotion, and the e-SLAM LiDAR sensor software. The manuscript is divided into the following sections. Section 2 describes an overview of the e-SLAM method. In Section 3, the principle of the path planning is presented. Subsequently, the motion interpolation for motion control is discussed in Section 4. Section 5 describes the discrete control scheme of the IMR, and the implementation as well as experimentation performed for method validation are presented in Section 6. Subsequently, the experimental results are discussed in Section 7 along with an insight into the integration of various pieces of software. Finally, we end with the conclusion in Section 8.

## 2. Exploration-Based SLAM (e-SLAM) Method

**Figure 1:**
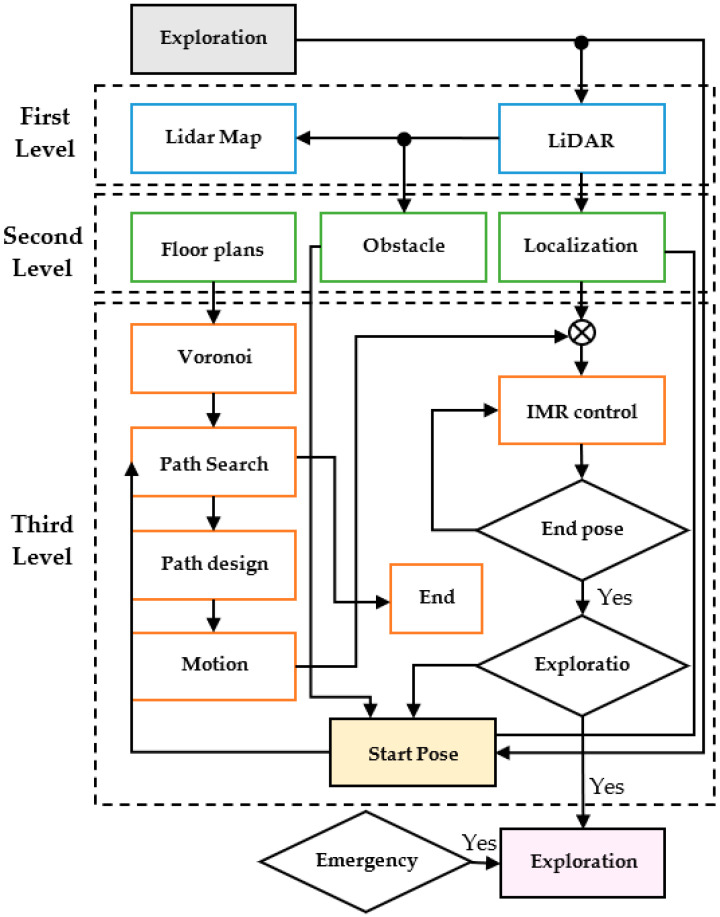
The e-SLAM scheme.

The exploration-based SLAM [2] was used to construct the map of an explored floor on smooth pavement using LiDAR. The current research work is to achieve the automatic exploration process for the indoor mobile robot (IMR) to fill out the missing pieces from the puzzle map. SLAM consists of three major stages: mapping, location, and path planning. In Figure 1, the e-SLAM is considered with a protocol of three levels. The top level includes LiDAR and mapping which is achievable by integrating LiDAR data into useful wall segments partitioning the building indoor space. These walls divide the space into rooms, utility spaces such as elevators, waiting space corridors, and the lobby. Next, there is a localization layer which is fed consecutively with LiDAR data. To achieve localization, e-SLAM instilled a constraint of using only LiDAR sensors. An IMU for the data fusion may be used to improve the accuracy of the positioning as well as the IMR direction, for experimental purposes only. However, in practice, the IMU may differ in accuracy from the LiDAR, due to the gyro drift and accelerometer noise. On the contrary, the IMU provides data with a much higher frequency, typically 500 Hz, than the LiDAR system does, which is typically 10 Hz. The third level consists of path planning which guides the IMR with its LiDAR to explore various segments in the indoor space. Certain difficulties are bound to be encountered by the IMR during the exploration process, such as narrower doors for it to pass through, and also a passerby may cause the IMR to go into an emergency stop. In this study, we attempt to avoid these difficulties by shutting the doors which do not need to be opened and by having guards simultaneously with the IMR for diverting passersby. The unexplored floor will be treated as a rectangular maze for the IMR to memorize the map and move on to its right side against the walls. The experiment is achieved inside the engineering school building with a 4000 m^2^ total floor area including labs, corridors, elevator waiting space, and the lobby.

**Figure 2:**
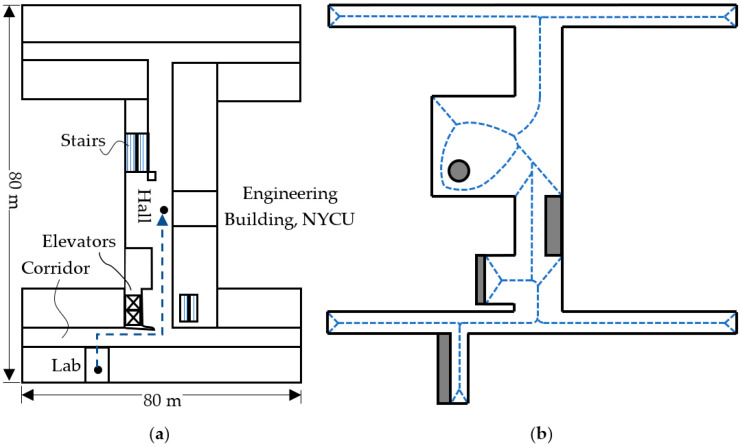
(**a**) Computed Voronoi segments of the floor map and (**b**) path search along the Voronoi edges.

This research deals with the path planning and its detailed motion command generation. It is assumed that the convex hull can be simplified into the Voronoi lines between walls as shown in Figure 2b. The vertices and edges have been calculated from the corresponding algorithms at the localization level. The path planning level starts with known vertices and generates the path from its current position to its next position which is defined by Voronoi vertices. The path search is based on following the walls on the right-hand side of the IMR. It is an analogy for the maze solver that keeps the right hand touching the wall in order to form a depth-first search algorithm for the tree search to come out from the maze. In our study, due to the IMR operating inside the building, the depth-first algorithm can be used to scan most of the space if the walls are connected. However, in some buildings, the rooms can be partitioned inside of the floor area. This can be deemed as several separate trees or a forest, in which the IMR will have to explore each different tree in a different run. For instance, purple thinner lines indicate a different tree with the walls disconnected from the black thicker lines.

## 3. Path Planning

The IMR moves on the floor on even pavement, and its trajectory is defined by a series of Voronoi vertices. The vertices forming a piecewise continuous polyline called the Voronoi path embody the tangential information of the IMR navigation with vertex (x,y). The corners formed by the vertices shall be provided with the minimum turn diameter *D* and the speed limit as well. Therefore, the Voronoi vertex may be in the following form:

(1) $$V=\left[\begin{array}{c} x\\ y\\ D \end{array}\right]$$

Two vertices are joined using an edge called the Voronoi edge E. The edges from a Voronoi path. A pose P of the electric vehicle consists of a 2D position and velocity with direction θ and speed v as follows:

(2) $$P=\left[\begin{array}{c} x\\ y\\ \theta \\ v \end{array}\right]$$

The pose can be shown graphically with the length of an arrow indicating the speed of the pose and the direction of the velocity.

### 3.1. Floor Voronoi Section and Path Search

The floor map is divided into distinct convex polygons according to the LiDAR data, and no three vertices of the polygon are set to be collinear. The Voronoi segments are computed as the bisectors bisecting the shortest perpendicular line between two walls or objects. Figure 2a depicts a schematic plan of an initially given floor, and the subsequent Voronoi segments are depicted in Figure 2b. A set of Voronoi vertices with their connecting edges can be written into a coincidence matrix as follows:

(3) $$A=\begin{array}{c} \begin{array}{c} V_{1}\\ \vdots \\ V_{n} \end{array} \end{array}\begin{array}{c} \begin{array}{ccc} V_{1} & \cdots & V_{n} \end{array}\\ \left[\begin{array}{ccc} 0 & \cdots & 0\\ \vdots & a_{i,j} & \vdots \\ 0 & \cdots & 0 \end{array}\right] \end{array}$$

In the adjacency matrix, the entry is of $a_{i,j}=e_{i,j}$ when there is an edge connecting vertices $V_{i}$ with $V_{j}$ and $a_{i,j}=0$ when either i=j for self-looping or there is no edge between $V_{i}$ and $V_{j}$. The paths from a specific vertex, say *k*, to another vertex, say *m*, can be obtained from the matrix multiplication when k≠m as follows:

(4) $$\begin{array}{c} \begin{array}{c} 1\\ \vdots \end{array}\\ m-1\\ \begin{array}{c} m\\ \begin{array}{c} \begin{array}{c} m+1\\ \vdots \end{array}\\ m \end{array} \end{array} \end{array}\left[\begin{array}{c} \begin{array}{c} \begin{array}{c} \cdots \\ \vdots \end{array}\\ \cdots \end{array}\\ \sum_{q}p_{k,m}\\ \begin{array}{c} \cdots \\ \begin{array}{c} \vdots \\ \cdots \end{array} \end{array} \end{array}\right]=A^{n}\left[\begin{array}{c} \begin{array}{c} \begin{array}{c} 0\\ \vdots \end{array}\\ 0 \end{array}\\ 1\\ \begin{array}{c} 0\\ \begin{array}{c} \vdots \\ 0 \end{array} \end{array} \end{array}\right]\begin{array}{c} \begin{array}{c} 1\\ \vdots \end{array}\\ k-1\\ \begin{array}{c} k\\ \begin{array}{c} \begin{array}{c} k+1\\ \vdots \end{array}\\ n \end{array} \end{array} \end{array}$$

There may be multiple paths, say *q*, going from vertex *k* to vertex *m*, and each of these paths has exactly *n* edges. The number *n* may range from 1 to the total number of vertices until the minimum distance or other optimality criteria are satisfied. The identified optimal Voronoi path can be further subdivided into dyads of Voronoi edges. A dyad may or may not join two coincident edges for the IMR to navigate on. The center point of those edges is calculated to confirm several consecutive 2D poses and directions for IMR movement. The novelty of this path design approach is the most minimal number of poses to designate a complete trajectory of the IMR’s path. Owing to the IMR’s inability to make a zero-radius turn at the coincident vertex, a smooth transition curve is required for the IMR to be precisely instructed and controlled accordingly. The design of the transition curve depending on how two consecutive edges coincide is discussed in the following path design section.

### 3.2. Path Design

**Figure 3:**
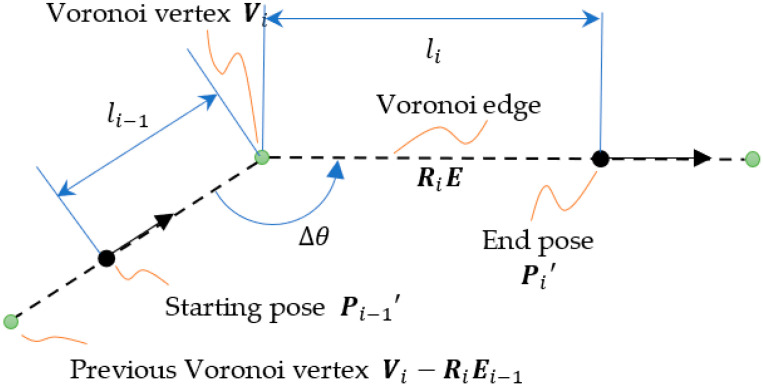
The Voronoi edges after rotation.

For two connected Voronoi edges $E_{i}$ and $E_{i-1}$, the EV navigates from a pose $P_{i-1}$ on the Voronoi edge $E_{i-1}$ to the other pose $P_{i}$ on $E_{i}$. The distance between $P_{i}$ and $V_{i}$ is denoted by $l_{i}$. In order to simplify the derivation, we first rotate the edges about the center, which is the Voronoi vertex $V_{i}$. The rotation with a two-dimensional rotation matrix $R_{i}$ makes the Voronoi edge $E_{i}$ align with the horizontal axis as shown in Figure 3. The pose after a rotation is expressed as follows:

(5) $$R_{i}⊗P_{i-1}=\left[\begin{array}{c} x^{\prime }\\ y^{\prime }\\ -∆\theta \\ v_{i-1} \end{array}\right]$$

The intersection angle of the Voronoi edges is ∆θ, and the rotational matrix is defined as follows:

(6) $$\begin{array}{c} {∆\theta =\theta_{i}-\theta }_{i-1}\\ \left[\begin{array}{c} x^{\prime }\\ y^{\prime } \end{array}\right]=R_{i}\left[\begin{array}{c} x\\ y \end{array}\right]=\left[\begin{array}{cc} c\theta_{i} & -s\theta_{i}\\ s\theta_{i} & c\theta_{i} \end{array}\right]\left[\begin{array}{c} x\\ y \end{array}\right] \end{array}$$

Assuming the Voronoi vertex $V_{i}$ has a minimum turn diameter D, we can then define the limiting curve in terms of the complementary angle $\varphi_{i}$ of the Voronoi edges.

(7) $$\varphi_{i}=\pi -∆\theta$$

**Figure 4:**
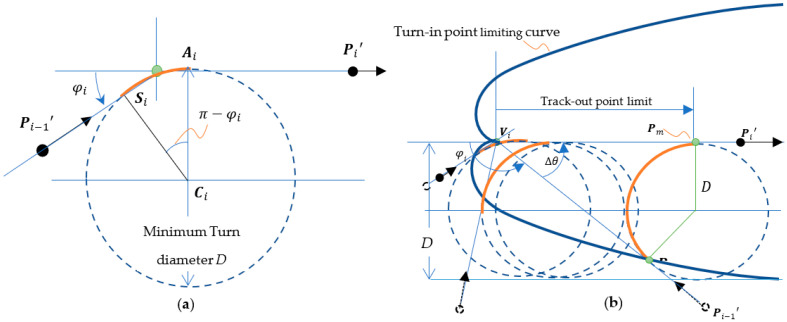
(**a**) Minimum turn diameter *D* and (**b**) the critical turn-in and track-out points of cornering motion.

According to the minimum turn diameter D as shown in Figure 4a, the center of cornering motion ${C_{i}}^{\prime }$ can then be derived as follows:

(8) $${C_{i}}^{\prime }=V_{i}+\frac{D}{2}\left[\begin{array}{c} tan\frac{\varphi_{i}}{2}\\ -1 \end{array}\right]$$

As shown in Figure 4b, one limitation, called the turn-in point limit, on the distance for the IMR between the pose of ${P_{i-1}}^{\prime }$ and the Voronoi vertex $V_{i}$ making a simple turn to approach the subsequent pose ${P_{i}}^{\prime }$ can be derived as follows:

(9) $${\left(\frac{2l_{i-1}}{D}\right)}^{2}<{\left(sin\varphi_{i}-tan\frac{\varphi_{i}}{2}\right)}^{2}+{\left(1-cos\varphi_{i}\right)}^{2}$$

The other limitation, called the track-out point limit, is the minimum distance for the IMR between the pose of ${P_{i}}^{\prime }$ and the Voronoi vertex $V_{i}$ and can also be determined as follows:

(10) $$\frac{2l_{i}}{D}>tan\frac{\varphi_{i}}{2}$$

As shown in Figure 4a, the turn-in point limiting curve has a trace curve $P_{t}$ derived as follows:

(11) $$P_{t}=V_{i}+X_{m}\left[\begin{array}{c} c∆\theta \\ s∆\theta \end{array}\right]$$

$P_{m}(x_{i}+X_{m},0)$ denotes the critical track-out point which is the endpoint of the cornering arc, which satisfies the condition as follows:

(12) $${2X}_{m}=D\cot\frac{\varphi_{i}}{2},-\pi <\varphi_{i}\leq \pi$$

When the dyad of edges satisfies both the turn-in point and track–out point limit, we can apply a minimum diameter turn on the locations of critical turn-in point $P_{t}$ and critical track-out point $P_{m}$ individually. This operation is referred to as the normal cornering motion. The EV path according to the Voronoi path is decomposed into a series of G codes in which G01 stands for a linear segment, G02 denotes a clockwise circular segment, and G03 denotes a counterclockwise segment. All G01, G02, and G03 segments are assigned with the turn-in and track-out poses for IMR navigation. As shown in Figure 4b, the path between ${P_{i-1}}^{\prime }$ and $P_{t}$ is a G01 motion. The path between $P_{t}$ and $P_{m}$ is a G02 motion. Due to the discrete control requirement, we need to further subdivide the path segments into discrete poses as the IMR motion command on each time tick.

### 3.3. Special Case for Path Design

**Figure 5:**
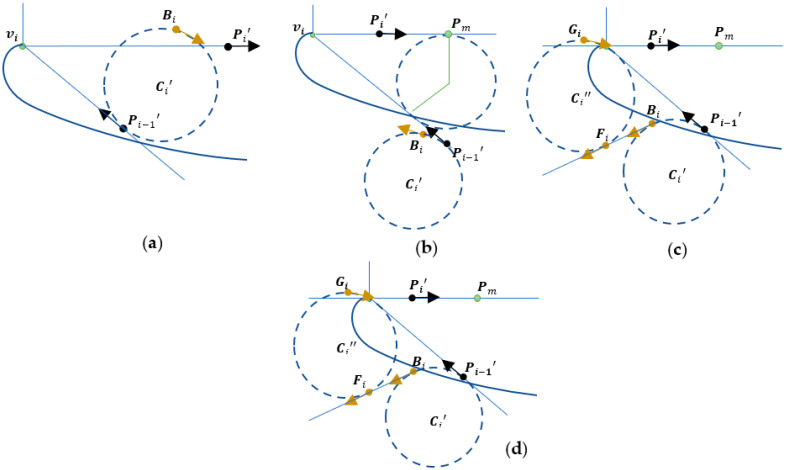
Special cases: (**a**) Rule 2 cornering motion, (**b**) Rule 3 cornering motion, (**c**) Rule 4 cornering motion, and (**d**) lane-change motion.

When any of the edges does not satisfy the turn-in or the track-out limitations, an extra path has to be generated, as discussed in the following:

1. Turn-in point limit fails when track-out point limit is satisfied: As shown in Figure 5a, we may find an intermediate pose $B_{i}$ above ${P_{i}}^{\prime }$ to first achieve the G02/G03 cornering motion from ${P_{i-1}}^{\prime }$ to $B_{i}$ and proceed to a normal cornering motion from $B_{i}$ to ${P_{i}}^{\prime }$ decreasing the complementary angle $\varphi_{i}$.
2. Turn-in point limit is satisfied when track-out point limit fails: As shown in Figure 5b, we may find an intermediate pose $B_{i}$ to first achieve the G03/G02 cornering motion from ${P_{i-1}}^{\prime }$ to $B_{i}$ and proceed to a normal cornering motion from $B_{i}$ to ${P_{i}}^{\prime }$ increasing the complementary angle $\varphi_{i}$.
3. Both the turn-in point limit and the track-out point limit fail: As shown in Figure 5c, we may find the set of intermediate poses $B_{i}$, F, and $G_{i}$ that first achieve the G03/G02 cornering motion from ${P_{i-1}}^{\prime }$ to $B_{i}$ connecting by a G01 linear motion from $B_{i}$ to $F_{i}$ and proceed to G02/G03 cornering motion from $F_{i-1}$ to $G_{i}$ followed by a normal cornering motion from $G_{i}$ to ${P_{i}}^{\prime }$.
4. The complementary angle $\varphi_{i}$ is very small: As shown in Figure 5d, if the adjacent vertices $B_{i}$ and ${P_{i}}^{\prime }$ are nearly collinear, then the normal cornering motion may still fail. In this case, we can determine the inflection point $I_{i}$ to achieve a lane-change motion which consists of one G03/G02 motion followed by the other G02/G03.

During handling the special cases, the numerical nesting loops are necessary to search for intermediate points, whereas in Rule 2 and Rule 3 cornering motion, there is only a single loop for the search. If the variation in IMR turn diameter is also concerned then there may be multiple nesting loops. For Rule 4 cornering motion, there are minimally two nesting loops when the two circles with centers ${C_{i}}^{\prime }$ and ${C_{i}}^{\prime \prime }$ are joined together. The evaluation function must be made efficient for real-time processing.

## 4. Motion Interpolation

**Figure 6:**
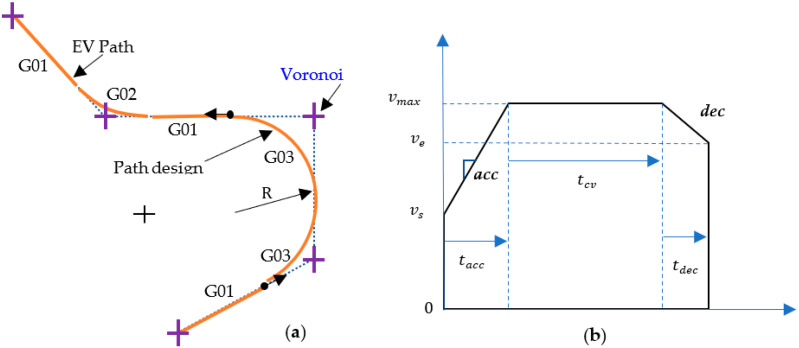
(**a**) Conversion of Voronoi path to motion path; (**b**) acceleration and deceleration for linear interpolation.

As shown in Figure 6a, all G01, G02, and G03 segments will be subdivided into fine G01 segments; the distances of each of the two subdivision poses $P_{k}$ and $P_{k-1}$ are governed by the equation as follows:

(13) $$v_{k}=\frac{d_{k}}{T}$$

where

$$d_{k}=\sqrt{{\left(x_{k}-x_{k-1}\right)}^{2}+{\left(y_{k}-y_{k-1}\right)}^{2}}$$

T denotes the sampling time for the discrete control. $v_{k}$ denotes the velocity at the k-th tick count which follows the motion interpolation and breaks down the G01 using the acceleration/deceleration method, introduced in the following sections.

### 4.1. Linear Segment Interpolation

There are two poses, referred to $P_{s,i}$ and $P_{e,i}$, used for specifying the linear segments. There is still one more pose ahead of $P_{e,i-1}$ that needs to be specified. In addition to the speed constraint $v_{max}$ which must be satisfied over the entire path from the movement authority, the acceleration ***acc*** and deceleration ***dec*** from the limitations of the IMR are the main concerns in the minimum time control. As shown in Figure 6b, the linear segment starts from the calculation of the acceleration and deceleration time as follows:

(14) $$t_{acc}=\frac{v_{i,max}-v_{s}}{acc}$$

(15) $$t_{dec}=\frac{v_{i,max}-v_{e}}{dec}$$

The total translation required by the acceleration and deceleration is derived as follows:

(16) $$d_{acc+dec}=\frac{{v_{i,max}}^{2}-{v_{s}}^{2}}{2acc}+\frac{{v_{i,max}}^{2}-{v_{e}}^{2}}{2dec}$$

where $d_{i}$ denotes the Euclidean distance between $P_{s,i}$ and $P_{e,i}$.

If the translation derived above is higher than the distance $d_{i}$ then we need to compute the feasible maximum velocity on the corresponding G01 path.

(17) $${\tilde{v}}_{i,max}=\sqrt{2(d_{i}+\frac{dec\cdot {v_{s}}^{2}+acc\cdot {v_{e}}^{2}}{acc+dec}})$$

With the feasible maximum velocity, we can recalculate the acceleration time as well as the deceleration time from Equations (15) and (16) and further to the constant velocity time as follows:

(18) $$t_{cv}=\frac{\left(d_{i}-d_{acc+dec}\right)}{v_{i,max}}$$

The time shown in Figure 6b is the local time starting from zero, which has to be converted into the tick counts once the paths are connected.

### 4.2. Arc Segment Interpolation

It is preferred for the IMR to traverse using a constant speed on the arc segment. However, the speed is constrained to go with a centrifugal force less than a limit. This limit can come from the slip angle of the IMR steering wheel or the jerk limit of the IMR motion. Both the centrifugal force and the jerk are a function of traversing speed and the turn radius. The speed of the arc segment may be governed as follows:

(19) $$constant=\frac{{v_{i,max}}^{n+1}}{{\left(\frac{D_{i}}{2}\right)}^{n}}$$

The power *n* can be 1 or 2 to represent the centrifugal acceleration or the jerk index individual. The motion interpolation can simply divide the arc into subdivisions that maintain the constant speed $v_{i,max}$ according to Equation (13).

### 4.3. Transition Curve

The transition curve is used to join the different segments into a smooth velocity profile. It is analogous to the track transition curve which is a mathematically calculated curve on a section of highway in which a straight segment changes into an arc segment or an arc segment changes into another arc segment with a different center of the radius. It is designed to prevent sudden changes in centrifugal (or lateral) acceleration. In the plane from the top view, the start of the transition curve is at an infinite radius, and at the end of the transition, it goes to a finite radius the same as the radius of the arc segment. It so forms a spiral. In practices of discrete control, we will need only to blend the difference in the traction speed as well as the steering angle control commands at the end of the former section connecting the latter segment. Such transition curves with bilateral blending can automatically form a kind of spiral that provides a continuous lateral acceleration to the wheels.

## 5. IMR Discrete Control Scheme

### 5.1. Vehicle Command Generation

**Figure 7:**
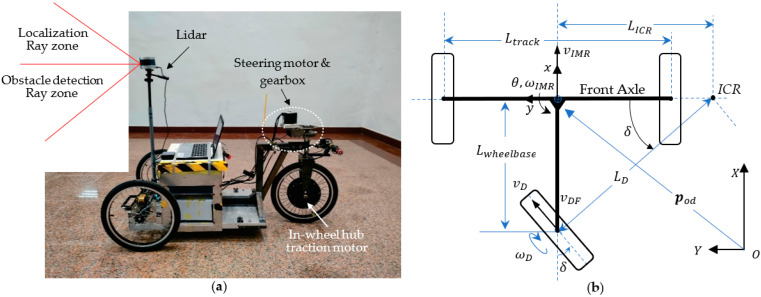
(**a**) The IMR configuration and (**b**) the body (or bogie) schematics of IMR.

Figure 7a shows the image of the IMR with a 1.5 kW in-wheel BLDC motor as the traction motor which powers the vehicle forward or backward, spins the wheel, and transmits power to the ground. This traction model is used for a battery-powered electric vehicle where the gearbox, clutch, transmission shaft, universal joint, and anti-roll bar are no longer used. The LiDAR is fitted at the front axle at a height of 180 cm from the ground level. The ray zones from the LiDAR are divided into two regions: the upper zone is used for the localization task, while the lower zone is used for the object detection task. Figure 7b shows the body (or bogie) schematics of the IMR which is a three-wheeled vehicle with an axle in front and a rear wheel on the back. The vehicle system parameters are given in Appendix A. The configuration of the IMR yields a rear-wheel drive and rear-wheel steering system. The terms denoted in the following equations are depicted in Figure 7b. The IMR motion may be simplified in the velocity equation for $v_{IMR}$ as follows:

(20) $$v_{IMR}=\omega_{D}r_{D}cos\delta$$

where $v_{IMR}$ denotes the velocity of the IMR. $\omega_{D}$ denotes the traction wheel speed. $r_{D}$ denotes the wheel radius of the traction wheel. δ denotes the steering angle. The instant center of rotation (ICR) is always on the extension line of the front axle. When the ICR is very far away from the vehicle, the IMR motion is subjected to a linear motion. When the ICR coincides with the center of the vehicle, which is the center of the front axle, the IMR is subjected to a pure rotation. The distance between the ICR and the center of the vehicle determines the instantaneous turn radius $L_{ICR}$. The radius $L_{ICR}$ is determined by the steering angle of the vehicle, which is as follows:

(21) $$L_{ICR}=\frac{L_{wheelbase}}{tan\delta }$$

The orientation θ of the IMR is determined by the integration of the rotation speed of the vehicle $\omega_{IMR}$ which is derived as follows:

(22) $$\omega_{IMR}=\omega_{D}\frac{r_{D}cos\delta }{L_{ICR}}=\frac{r_{D}}{L_{wheelbase}}\omega_{D}sin\delta$$

The discrete position of the vehicle is then formulated in the global coordinate system OXY as follows:

(23) $$P_{k}\equiv {\left[\begin{array}{c} x_{k}\\ y_{k}\\ \theta_{k}\\ v_{k} \end{array}\right]}_{IMR}=\left[\begin{array}{c} x_{k-1}+cos\theta_{k-1}\cdot \omega_{D,k}\cdot r_{D}\cdot cos\delta_{k}\cdot T\\ y_{k-1}+sin\theta_{k-1}\cdot \omega_{D,k}\cdot r_{D}\cdot cos\delta_{k}\cdot T\\ \theta_{k-1}+\frac{r_{D}\cdot T}{L_{wheelbase}}\omega_{D,k}\cdot sin\delta_{k}\\ \omega_{D,k}\cdot r_{D}\cdot cos\delta_{k} \end{array}\right]$$

It is an optimization problem from one pose $P_{k-1}$ of the IMR to the other pose $P_{k}$, which has four equations to fulfill with only two control inputs $\omega_{D,k}\equiv \omega_{D}(k)$ and $\delta_{k}\equiv \delta (k)$ in the time tick k. Assuming the pose estimation is ${\tilde{P}}_{k-1}$ which may be obtained from the sensor feedback using LiDAR and/or IMU with Kalman filtering [2], the control of $\omega_{D,k}$ and $\delta_{D,k}$ to achieve a new pose $P_{k}$ is derived with the following linearization as:

(24) $$\begin{array}{c} cos\delta_{k}\approx cos\delta_{k-1}-sin\delta_{k-1}\cdot ∆\delta \\ sin\delta_{k}\approx sin\delta_{k-1}∆\delta +cos\delta_{k-1} \end{array}$$

where

$$∆\delta =\delta_{k}-\delta_{k-1}$$

The associated discrete equation of control can be written as follows:

$$B\left[\begin{array}{c} ∆\omega \\ ∆\delta \end{array}\right]=b$$

where

(25) $$\begin{array}{c} ∆\omega =\omega_{D,k}-\omega_{D,k-1}\\ B=\left[\begin{array}{cc} \frac{\alpha_{k-1}c\theta_{k-1}}{\omega_{D,k-1}} & -\beta_{k-1}c\theta_{k-1}\\ \frac{\alpha_{k-1}s\theta_{k-1}}{\omega_{D,k-1}} & -\beta_{k-1}s\theta_{k-1}\\ \frac{\alpha_{k-1}}{L_{wheelbase}\omega_{D,k-1}} & \frac{\beta_{k-1}}{L_{wheelbase}}\\ c\delta_{k-1}r_{D} & -\omega_{D,k}s\delta_{k-1}r_{D} \end{array}\right] \end{array}$$

$$b=P_{k}-({\tilde{P}}_{k-1}+\left[\begin{array}{c} \alpha_{k-1}c\theta_{k-1}\\ \alpha_{k-1}s\theta_{k-1}\\ \frac{\alpha_{k-1}}{L_{wheelbase}}\\ \alpha_{k-1}-v_{k-1} \end{array}\right])$$

$$\alpha_{k-1}=r_{D}T\omega_{D,k-1}c\delta_{k-1}$$

$$\beta_{k-1}=r_{D}T\omega_{D,k-1}s\delta_{k-1}$$

In mathematics, a system of equations is considered to be overdetermined if the number of equations is more than the unknowns. The method of ordinary least squares can be used to find an approximate solution to overdetermined systems. With this formula, an approximate solution is found when no exact solution exists, and it gives an exact solution when one does exist. A similar method has been used by Antonelli et al. [34]. Equation (20) can be solved in the least square form based on the proportional-derivative control law as follows:

(26) $$\left[\begin{array}{c} ∆\omega \\ ∆\delta \end{array}\right]=\left[\begin{array}{c} \omega_{k}\\ \delta_{k} \end{array}\right]-\left[\begin{array}{c} \omega_{k-1}\\ \delta_{k-1} \end{array}\right]={\left(B^{T}B\right)}^{-1}B^{T}\left[\begin{array}{cccc} K_{p\_pos} & 0 & 0 & 0\\ 0 & K_{p\_pos} & 0 & 0\\ 0 & 0 & K_{p\_angle} & 0\\ 0 & 0 & 0 & K_{p\_vel} \end{array}\right]b$$

$K_{p\_pos},\;K_{p\_angle},\;and\;K_{p\_vel}$ stand for the proportional gain of the IMR position, orientation, and velocity as estimated by the LiDAR sensor. A certain state-observer-based control [35] could be used in the control system when the dynamic model is known [36]. We performed the state space control as stated in Equation (26) with a sampling frequency of 10 Hz, which is much lower than the sampling frequency of 200 Hz required in a typical autonomous car. The low sampling frequency is due to the LiDAR localization computation for the LiDAR map exploration purpose. The state space control is focused on robustness instead of precision. The traction control $\Omega_{k}$ for $\omega_{k}$ and the steering angle control ${∆}_{k}$ for achieving $\delta_{k}$ in the individual motor driver subsequently use the PID control scheme with 100 Hz control as follows:

(27) $$\left[\begin{array}{c} \Omega_{k}\\ {∆}_{k} \end{array}\right]=K_{P}\left[\begin{array}{c} \omega_{k}-\omega_{k,sensor}\\ \delta_{k}-\delta_{k,ensor} \end{array}\right]+K_{I}\int \left[\begin{array}{c} \omega_{k}-\omega_{k,sensor}\\ \delta_{k}-\delta_{k,ensor} \end{array}\right]dt+K_{d}\frac{d}{dt}\left[\begin{array}{c} \omega_{k}-\omega_{k,sensor}\\ \delta_{k}-\delta_{k,ensor} \end{array}\right]$$

There is still a nonlinear effect due to the current limit and speed limit related to the rated torque and rated speed of the motors of the IMR, which can only be verified in an experiment.

### 5.2. LiDAR-Based Image Segmentation

The LiDAR image-based localization of the IMR uses the e-SLAM method [2], which includes the following procedures:

1. Determining the initial rotation using the minimum bounding box method.
2. Forming the LiDAR meshes for the walls vertical to the floor.
3. Determining the most significant corner (MSC) from the vertical walls based on the histogram analysis.
4. According to the base LiDAR pose frame, referred to as the floor LiDAR origin, performing the inverse transformation to map all LiDAR data back to the room map.
5. According to the rotation and translation of the LiDAR pose scheme relative to the floor LiDAR origin, performing the active localization.
6. According to the least quadratic estimation (LQE) method associated with the IMR steering and traction control as stated in Equation (26), performing the localization estimation.
7. According to the LiDAR data XY projection image, performing the IMR translation update.
8. Performing the room segmentation and floor management.

**Figure 8:**
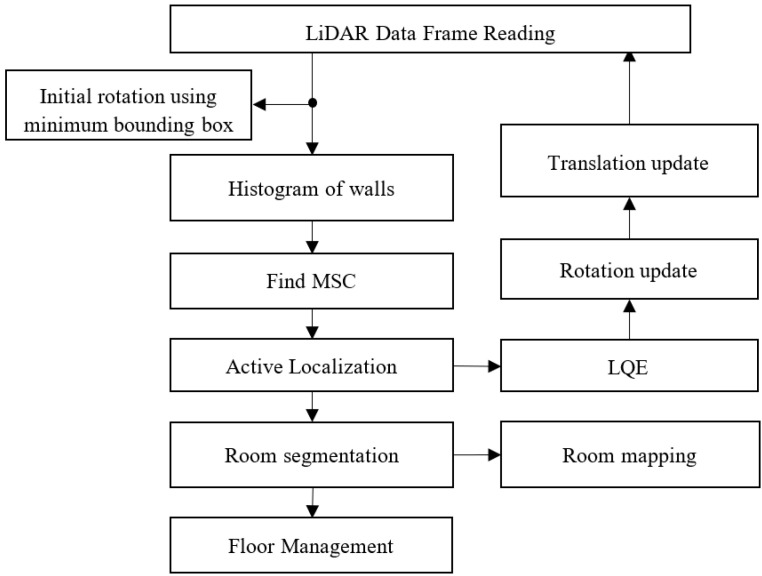
Flowchart of e-SLAM.

**Figure 9:**
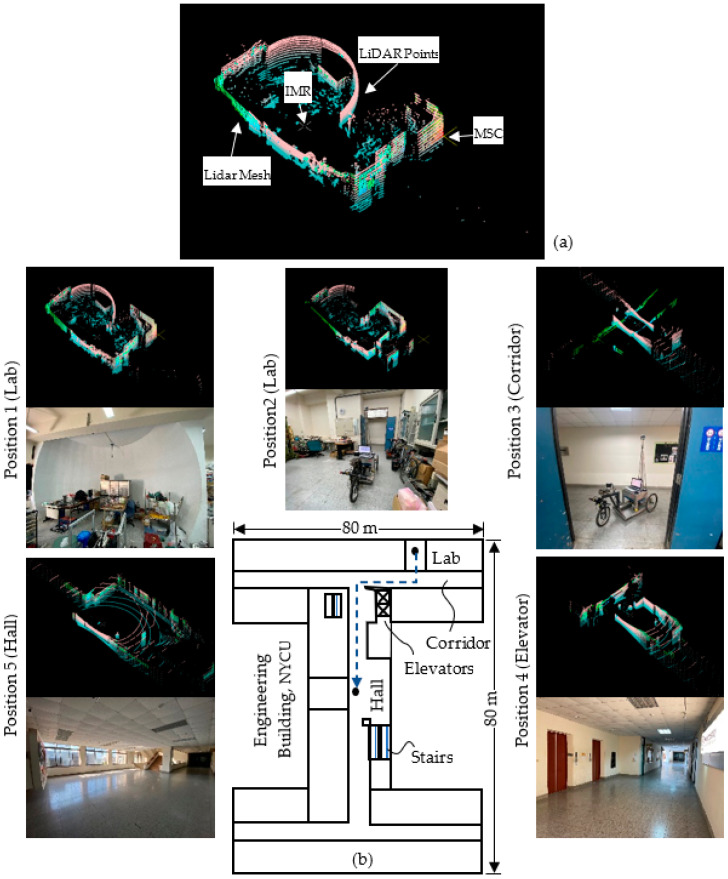
The LiDAR-based localization: (**a**) LiDAR data, (**b**) floor map and LiDAR map.

The software is implemented using a VLP_16 LiDAR with 16 rings made by Velodyne Inc. (San Jose, CA, USA). A remarkable contribution in IMR LiDAR localization in [2] consists in the fact that the authors use solely a single sensor source, i.e., LiDAR, for indoor exploration and localization of the robot. A flowchart of the e-SLAM algorithm is shown in Figure 8. The LiDAR is positioned onto the center of the front axle of the IMR with a total height of 180 cm from the ground as shown in Figure 7a. The resulting LiDAR images are shown in Figure 9a. The unprocessed LiDAR data are acquired from the Socket communication associated with VLP_16 devices. The LiDAR data are then reorganized into the mesh image for the construction of the vertical walls which are subsequently used to form the LiDAR pose frame. The contemporary most significant corner (MSC) is the origin of the LiDAR pose frame. The location of the IMR is shown in Figure 9a using a cross. We chose the top portion of the LiDAR rings at 1, 3, 5, 7, 9, 11, 13, and 15 degrees to form the wall. The frame rate is around 5–10 data frames per second for updating the IMR location. The total distance accumulated during the navigation is about 45 m. The maximum speed of the IMR is 200 cm/s, which is a medium speed in IMR applications. With the obstacle detection using the LiDAR rings at −1, −3, −5, −7, −9, −11, −13, and −15 degrees, the IMR comes to a halt due to passersby. At a height of 180 cm from the ground level, the LiDAR can detect obstacles more than 160 cm from the ground and 80 cm apart from the vehicle. Keeping the LiDAR sensor below 180 cm provides noise while collecting point clouds due to which the LiDAR localization algorithm fails to provide a reference point, i.e., the most significant corner (MSC).

The test environment was Engineering Building V, an I-shape building with two wings and a connection section, in the Guang-Fu Campus of the NYCU. The experiment was performed on the fifth floor with a floor area of around 5000 m^2^; the top view is shown in Figure 9b. The wall material is made from concrete and white fiber cement layered onto the wall surface with a solar reflectance of 0.40. In the lab, there is a dome screen made of wood, which occupies one-quarter of the lab space. There is also a mirror on the wall adjacent to the door which can produce fake feature data by reflecting the laser light.

The current pose ${\tilde{P}}_{k-1}$ in (25) is updated in 5 to 10 Hz from the LiDAR-based localization. The actual steering and traction control are performed using a 20 Hz traction velocity and steering angle update using Equation (26).

## 6. Implementation and Experiment

WINPC32, a programmable control and HMI platform developed by Hurco Automation Ltd., was implemented for the IMR controller. We implemented our IMR control for both the steering angle and traction velocity using a Graphics Program Editor and external C. The real-time data between different software used a global data exchange (GDS) with a data refresh rate of 20 Hz. An emergency stop button was used to terminate the control and bring the IMR to a still position within 500 ms. The acceleration and deceleration profiles were also specified according to the motor capability and accuracy. The sensor compensation could also be implemented using the global data exchange to calibrate the errors found, such as the potential meter alignment for the steering angle control.

### 6.1. Sidomotion Software

**Figure 10:**
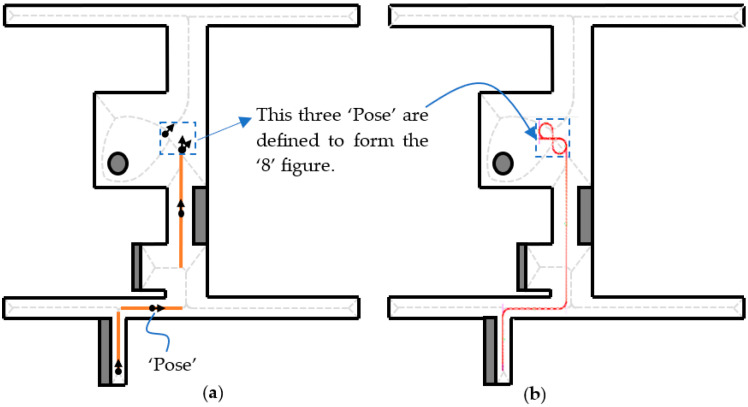
Path planning using Sidomotion (**a**) pose information; (**b**) designed path according to the pose information.

The Sidomotion software is implemented in VC6.0 according to Section 3 and Section 4. The software takes consecutive pose information (position and orientation) shown in Figure 10a as input. The intermediate vertices at the corner are then calculated to design the circular motion. The two pieces of pose information shown in the blue dotted squares are responsible for the “8” figure motion in Figure 10b. The red circles are the interpolation positions according to the linear and corner turning velocity input in the controller stated in the previous section. A dense sighting of the red circles at the corner turning depicts a slow velocity while on a linear motion; the far-placed red circles show an increase in velocity. The adjacent vertices are joined using the edge definition. One has to determine the best trajectory among the multiple trajectories between the starting and the destination vertex.

In this example, we can find a path, providing the starting vertex is “0” and the destination vertex is “10”, by calling the “FindPath” function. We can also specify the “0” in the “FindPoseFromArrayPath” function to indicate that the first trajectory found from the depth-first search is used. The green circles are the middle points (“Pose”) of the adjacent edges, which are the highest velocity positions at different edges. The current IMR position as well as the steering angle and the traction speed derived from Equation (26) are simultaneously shown in the same window.

### 6.2. LiDAR Localization Software

**Figure 11:**
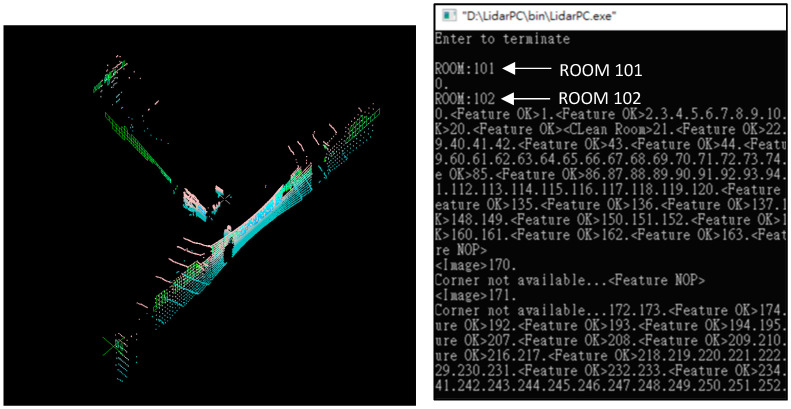
LiDAR localization software.

The LiDAR localization is a console-based software due to the communication port of Velodyne VLP_16 using “ws2_32.lib”. This causes a breakpoint between the Sidomotion and the LiDAR software; therefore, we need the GDS from the WINPC32 to join the information together. The message along with the LiDAR image is streaming on the console screen. As shown in Figure 11, the LiDAR software outputs ROOM101, ROOM102, ROOM103, etc., which are sent to the floor manager to distinguish different rooms identified during the IMR navigation. The message “Feature OK” indicates the most significant corner (MSC) that changes from one wall corner to the other when the visibility of the walls changes. The accumulation for the MSC displacement is taken into account for the IMR translation.

Whenever the “Feature NOP” is sent out, the localization based on the MSC information is no longer valid and the image comparison process is activated. The image comparison is to compare the current LiDAR data points to the LiDAR mesh stored in the memory to calculate the location and orientation of the IMR. The MSC information is not valid in many situations, including (1) the blockage of the LiDAR rings by a passerby, (2) a lengthy corridor which results in the LiDAR rays failing to find the horizontal wall, and (3) the processing time of all software being too long and delaying the Socket communication of the LiDAR. The remedies slow down the linear as well as the corner turning velocities to allow the LiDAR data to present a clear image of the walls as well as a comparison to the existing LiDAR meshes.

### 6.3. Experiment

**Figure 12:**
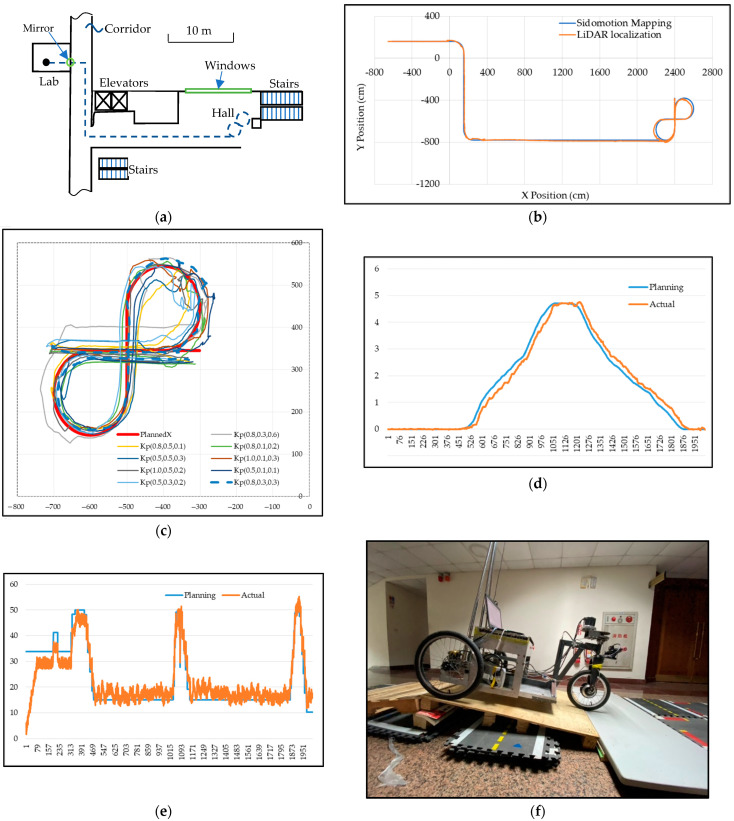
IMR navigation control: (**a**) floor plans of Engineering Building 5th Floor, NYCU; (**b**) path planning experiment data; (**c**) “8” figure test; (**d**) IMR orientation during navigation; (**e**) speed command sent to IMR according to Equation (26) compared with the trajectory velocity planning; (**f**) 10-degree ramp test.

The experiments were conducted after all sensor and actuator calibration [34] to ensure that all sensor and actuator parameters were at optimum settings. Due to the reflection of the LiDAR signal from either the mirror or the window glasses, there is a certain noise-to-signal ratio resulting from the LiDAR image-based localization. The control commands could be oscillating corresponding to the sensor noise. The IMR will also stop whenever a passerby is detected in front of the IMR. Any of the situations stated above can cause the IMR to deviate from the desired path. A simple way to filter out the noise is to map the current LiDAR pose back onto the desired path and recalculate the new control commands to navigate the IMR. The Sidomotion software maps the LiDAR localization pose of the IMR to the nearest position on the desired path (blue color line) as shown in Figure 12b; therefore, the IMR is commanded to follow the desired path whenever the IMR deviates from it.

A set of experiments was conducted to explore the parameter tuning of the control gain stated in Equation (26). Different arrangements of linear position, angular position, and velocity gain according to the orthogonal array from Taguchi experimental design with three levels and three factors were set up. The results having different “8” figures using different control gain settings are shown and compared in Figure 12c. It is found that the LiDAR sensor had imposed certain sensor errors on the top-right corner of the “8” figure. Taking into account the sensor error, we identify the optimal settings to be $K_{p\_pos}=0.8$, $K_{p\_angle}=0.3$, and $K_{p\_vel}=0.3$ with more than 95% confidence level. Using the optimal settings on the sensor control gains, the feedback control was performed, and the result is shown in Figure 12b.

The control command $P_{k}$ is generated according to the current pose ${\tilde{P}}_{k-1}$ calculated from the path point due to the Sidomotion mapping. There is a mirror in front of the gateway of the lab where the IMR makes a turn to the right into the corridor, which reflects the LiDAR rays, affecting the accuracy of the LiDAR localization. The windows as well as the stairs with complicated alignment can also affect the localization accuracy. Figure 12d shows the orientation difference between the command generated according to Equation (26) and the LiDAR localization data. Figure 12e shows the speed planning and control; the actual control is not smooth due to obstacle detection. The linear speed is planned at 50 cm/s while the cornering speed is 15 cm/s.

Both the LiDAR sensor and the motor control were tested on a ramp test with a 10° ramp angle as shown in Figure 12f. A 250 W hub motor was used for traction. This is a challenging task for a high inclination angle due to the weight of the IMR, which is around 45 Kg, which includes the battery with a gross weight of 30.35 kg. On a lengthy ramp, the IMR loses its resolution on the LiDAR sensor due to its close proximity to the ceiling which ultimately blocks the LiDAR rays.

## 7. Discussion

The factors that affect IMR navigation include the following:

(1) The vehicle speed planning including the maximum linear speed and the corner turning speed; the LiDAR localization can fail due to the speed command higher than 200 cm/s which is 8 kmph. In the 10 Hz sampling, the displacement of the IMR is 20 cm per frame, which may deteriorate the orientation judgment of the IMR localization. Therefore, it is recommended that the speed of 100 cm/s yields the best robustness.
(2) The height of the LiDAR; it is preferred to set it slightly higher than the average human height around 180 cm because of the upper eight rings and the lower eight rings of the LiDAR that are used to perform the IMR localization and to detect obstacles. To obtain a higher resolution of the IMR localization, we can also select nine upper rings for the localization and seven lower rings for obstacle detection. This situation can happen in the case of a long corridor where the horizontal wall is at a distance of 20 m away. On the 20 m away wall, the two-degree ring spacing is mapped into 70 cm height.
(3) The obstacle angle and distance; there is an oval cone looking front used to detect the obstacle, and the width of the cone is determined by the obstacle angle which is set to be ±15°. A larger obstacle angle can cause the IMR to be blocked by the side walls. The obstacle detection distance is recommended to be 80 cm.
(4) The Sidomotion command generation; the command always brings the IMR forward on the path, and thus we have to choose the number of interpolation points ahead on the path planned to bring the IMR forward. A command produced from a small increment from the current pose can slow down the IMR navigation; however, the IMR can lose control when the increment is large. Empirically, we will set the current IMR control command into the next time tick achievable, making it speed-dependent. For instance, the command position is 40 cm ahead of the current pose when the speed is 100 cm/s.

This research deals with the integration of various pieces of software, and hence a step-by-step integrating procedure includes the following:

  I. Floor manager, which converts the floor plan into a Voronoi map for the Sido (Service In Door Operation) motion service use.
 II. Service manager, which creates the Sido motion planning due to the assigned task.
III. Camera manager, which is not included in this paper.
IV. LiDAR manager, which sets up the LiDAR parameters and performs the e-SLAM localization.
 V. Motor manager, which sets up control parameters for both steering and traction motors and performs real-time communication with the EtherCAT motor driver.
VI. IMR HMI, which provides the human–machine interface to the user.

The software is currently integrated using VS2019 to consolidate the projects individually developed in VC6.0 and VS2010. The conversion process requires the CMAKE with CMakelists.txt to help the transition from VC6.0 compiler projects into the VS2019 IDE project assisted by BCGSoft GUI tools. The IMR HMI utilizes the WINPC32 development tool kit to generate HMI from the GPeX module, the sequential logic from the PLC module, and the real-time motion control from the MOTION module.

## 8. Conclusions

We presented a method to use an IMR not only as an e-SLAM tool but also as a specific functional vehicle, i.e., an automatic inter-office delivery robot using only LiDAR. Given an initial floor plan, the Voronoi edges are first computed using only LiDAR cloud points. To perform the path planning, a minimum number of pieces of pose information along the edges is used for path search, path design, and special design methods in order for the IMR to navigate an unknown floor. The motion interpolation is used to subdivide the Voronoi edges into small increment points for the IMR motion command generation. The task of motion interpolation is categorized into linear segment and arc segment interpolations depending on the linear and cornering velocity, acceleration, and deceleration limits, respectively. The current pose of the IMR is calculated from the LiDAR localization e-SLAM method presented in a previously published study. The pose including the location and orientation of the IMR is then mapped into the designated path, and the control command is generated according to the speed and the current pose of the IMR. The LiDAR data are not only used to generate the floor LiDAR map for space reconstruction but also used to perform obstacle detection on the objects which are present differently from the original floor plans. The implementation of the IMR with e-SLAM integrates four sets of software, namely the PLC-based controller platform WINPC32, the EtherCAT motor driver, the Path planning software Sidomotion, and the e-SLAM LiDAR sensor software. The experiment was performed using a portion of the floor exploration that was used to demonstrate the feasibility of the IMR system. This study initialized a possibility for the indoor service robot to be installed easily without the pre-scanning of any building. An “8” shape was added at the end of the experiment to prove that the IMR is equipped with conventional automobile control capability.

The drawbacks of the current e-SLAM implementation lie in the LiDAR alone for its complexity in large area exploration as the mapping loses its resolution for long distances. In order to solve that, camera image processing is still needed to reset the LiDAR origin from consecutive rooms to obtain a better resolution. The camera also needs to account for recognizing the obstacles detected by the LiDAR during exploration.

Much research work can be further implemented in the future, including the use of artificial intelligence (AI) to facilitate maze-like exploration and allow detours when a passer-by or obstacle is detected, the use of video images from the camera in combination with the LiDAR point cloud data together with the camera image to form a human-readable virtual reality, and the provision of an IMR service layout via smartphone queries.

## Data Availability

Data sharing is not applicable.

## Appendix A

Powertrain Systems	- 1.5 kW BLDC traction motor. - Single rear-wheel drive. - 20″ wheels with tube type (20 × 1.65).
Chassis and Steering Systems	- A ladder frame aluminum with steel backbone. - 1 × 24 V BLDC motor for steering position control.
Electronic Systems	- 1 × 24 V drive with EtherCAT communication protocol for steering. - 1 × 48 V drive for traction controls. - 1 × 45 Ah 48 V Li-ion battery for traction system. - 2 × 12 V battery for steering system. - 1 main controller with display.
Physical Specifications	- Vehicle dimensions: 1630 (L) × 750 (W) mm. - Wheel base: 1200 mm. - Wheel tread: 700 mm. - Vehicle height (without LiDAR): 860 mm. - Front end frame (including gearbox and steering motor): 10.7 kg.

## References

[1] Betz J., Wischnewski A., Heilmeier A., Nobis F., Stahl T., Hermansdorfer L., Lohmann B., Lienkamp M. (2018). What can we learn from autonomous level-5 motorsport?. Proceedings of the 9th International Munich Chassis Symposium.

[2] Ismail H., Roy R., Sheu L.J., Chieng W.H., Tang L.C. (2022). Exploration-Based SLAM (e-SLAM) for the Indoor Mobile Robot Using Lidar. Sensors.

[3] Bresson G., Alsayed Z., Yu L., Glaser S. (2017). Simultaneous Localization and Mapping: A Survey of Current Trends in Autonomous Driving. IEEE Trans. Intell. Veh..

[4] Mouragnon E., Lhuillier M., Dhome M., Dekeyser F., Sayd P. Real time localization and 3D reconstruction. Proceedings of the IEEE CVPR.

[5] Campos C., Elvira R., Rodríguez J.J.G., Montiel J.M.M., Tardós J.D. (2021). ORB-SLAM3: An Accurate Open-Source Library for Visual, Visual–Inertial, and Multimap SLAM. IEEE Trans. Robot..

[6] Cadena C. (2016). Past, Present, and Future of Simultaneous Localization and Mapping: Toward the Robust-Perception Age. IEEE Trans. Robot..

[7] Li P., Ke Z. Feature-based SLAM for Dense Mapping. Proceedings of the International Conference on Advanced Mechatronic Systems (ICAMechS).

[8] Hess W., Kohler D., Rapp H., Andor D. Real-time loop closure in 2D LIDAR SLAM. Proceedings of the IEEE International Conference on Robotics and Automation (ICRA).

[9] Schulz C., Zell A. Real-Time Graph-Based SLAM with Occupancy Normal Distributions Transforms. Proceedings of the IEEE International Conference on Robotics and Automation (ICRA).

[10] Menezes M.C. Automatic Tuning of RatSLAM’s Parameters by Irace and Iterative Closest Point. Proceedings of the IECON 2020 the 46th Annual Conference of the IEEE Industrial Electronics Society.

[11] Wu H. Extending RatSLAM Toward A Multi-Scale Model of Grid Cells. Proceedings of the 7th International Conference on Control, Automation and Robotics (ICCAR).

[12] Muraleedharan A., Okuda H., Suzuki T. (2022). Real-Time Implementation of Randomized Model Predictive Control for Autonomous Driving. IEEE Trans. Intell. Veh..

[13] Jigong L., Yiwei F., Chaoqun Z. A Novel Path Planning Method Based on Certainty Grids Map for Mobile Robot. Proceedings of the Chinese Control Conferenc.

[14] Paden B., Čáp M., Yong S.Z., Yershov D., Frazzoli E. (2016). A Survey of Motion Planning and Control Techniques for Self-Driving Urban Vehicles. IEEE Trans. Intell. Veh..

[15] Maurović I., Seder M., Lenac K., Petrović I. (2018). Path Planning for Active SLAM Based on the D* Algorithm with Negative Edge Weights. IEEE Trans. Syst. Man Cybern. Syst..

[16] Hoshi M., Hara Y., Nakamura S. (2022). Graph-based SLAM using architectural floor plans without loop closure. Adv. Robot..

[17] Gavshinde L., Singh A.K., Krishna K.M. Trajectory planning for monocular SLAM systems. Proceedings of the IEEE International Conference on Control Applications CCA.

[18] Zhang Y., Hu Y., Hu X., Xing B. Path Planning for Mobile Robot Based on RGB-D SLAM and Pedestrian Trajectory Prediction. Proceedings of the 4th Annual International Conference on Data Science and Business Analytics (ICDSBA).

[19] Choi H., Chae H.W., Song J.-B. Keyframe Tracking-Based Path Planner for Vision-Based Autonomous Mobile Robots. Proceedings of the 19th International Conference on Control, Automation and Systems (ICCAS).

[20] Feng J., Yang B., Pei X., Zhou P. Research on Path Planning and Control of Driverless Logistics Train. Proceedings of the 5th CAA International Conference on Vehicular Control and Intelligence (CVCI).

[21] Seder M., Petrovic I. Dynamic window based approach to mobile robot motion control in the presence of moving obstacles. Proceedings of the IEEE International Conference on Robotics and Automation.

[22] Yao M., Liu Y., Xu C. Locating, Mapping and Motion Planning of Smart Wheelchair. Proceedings of the 40th Chinese Control Conference (CCC).

[23] Qi Y., Xie B., Huang X., Yuan M., Zhu C. Path Planning of Mobile Robot Based on Improved Particle Swarm. Proceedings of the Chinese Automation Congress (CAC).

[24] Liu Y., Jiang L., Zou F., Xing B., Wang Z., Su B. Research on path planning of quadruped robot based on globally mapping localization. Proceedings of the 3rd International Conference on Unmanned Systems (ICUS).

[25] Liu Z. Implementation of SLAM and path planning for mobile robots under ROS framework. Proceedings of the 6th International Conference on Intelligent Computing and Signal Processing (ICSP).

[26] Talwar D., Jung S. Particle Filter-Based Localization of a Mobile Robot by Using a Single Lidar Sensor under SLAM in ROS Environment. Proceedings of the 19th International Conference on Control, Automation and Systems (ICCAS).

[27] Wei Y., Zhang H., Deng G., Zhong H., Liu L. Research on the SLAM of Mobile Robot Based on Particle Filter Method. Proceedings of the IEEE 9th Annual International Conference on CYBER Technology in Automation, Control, and Intelligent Systems (CYBER).

[28] Mukhtar H., Hasan M.A., Khan M.U.G. ROS-Based Global Path Planning for Autonomous Ground Robot Using the Pre-Built Map of the Environment. Proceedings of the International Conference on Robotics and Automation in Industry (ICRAI).

[29] Cheng Z., Li B., Liu B. Research on Path Planning of Mobile Robot Based on Dynamic Environment. Proceedings of the IEEE International Conference on Mechatronics and Automation (ICMA).

[30] Yang L., Gong J., Xiong G., Yang T., Wu M., Zhang S. Unmanned Vehicle Path Planning for Unknown Off-Road Environments with Sparse Waypoints. Proceedings of the IEEE Intelligent Transportation Systems Conference (ITSC).

[31] Zhang X., Lai J., Xu D., Li H., Fu M. (2020). 2D Lidar-Based SLAM and Path Planning for Indoor Rescue Using Mobile Robots. J. Adv. Transp..

[32] Moghadam P., Wijesoma W.S., Feng D.J. Improving path planning and mapping based on stereo vision and lidar. Proceedings of the 10th International Conference on Control, Automation, Robotics and Vision.

[33] Zhao S., Hwang S.H. Path planning of ROS autonomous robot based on 2D lidar-based SLAM. Proceedings of the International Conference on Information and Communication Technology Convergence (ICTC).

[34] Antonelli G., Chiaverini S., Fusco G. (2005). A calibration method for odometry of mobile robots based on least squares technique: Theory and experimental validation. IEEE Trans. Robot..

[35] Ismail H., Chieng W.H., Jeng S. (2021). A New Approach of Antiskid Braking System (ABS) via Disk Pad Position Control (PPC) Method. SAE Int. J. Commer. Veh..

[36] Ismail H., Chiang C., Chieng W.H. (2023). Onboard Sensor and Actuator Calibration of a Tripod Electric Vehicle Using Circular, Linear, and Cornering Motion Tests. SAE Int. J. Commer. Veh..

